# Special Issue: Molecular Therapeutic Strategies in Allergic Diseases

**DOI:** 10.3390/ijms27219507

**Published:** 2026-10-24

**Authors:** Emilia Majsiak, Natalia Łępa, Sylwia Smolinska-Wilczynska

**Affiliations:** 1 Department of Health Promotion, Faculty Health of Sciences, Medical University of Lublin, Staszica 4/6, 20-081 Lublin, Poland; emilia.majsiak@umlub.edu.pl; 2 Medical University of Lublin, Aleje Racławickie 1, 20-059 Lublin, Poland; 3 Department of Clinical Immunology, Faculty of Medicine, Wroclaw Medical University, Parkowa 34, 51-616 Wroclaw, Poland

## 1. Introduction

This Special Issue of the *International Journal of Molecular Sciences* brings together seven contributions addressing allergen identification, molecular allergy diagnostics (MAD), including component-resolved diagnostics (CRD), and therapeutic strategies for allergic diseases. Their shared concern is how increasingly detailed molecular information can improve the understanding and management of clinically heterogeneous disorders. Identifying the relevant trigger remains challenging when sensitization involves multiple allergen components, cross-reactive protein families, or unexpected allergen sources [1]. Molecular approaches help clarify these patterns, but their value depends on interpretation alongside the patient’s history and other diagnostic findings [1,2]. Allergen-specific IgE indicates sensitization rather than clinical allergy, and component-specific IgE concentrations do not reliably predict the severity of a future reaction. Likewise, qualification for allergen immunotherapy (AIT) requires a demonstrated relationship between exposure and symptoms, rather than a detailed sensitization profile alone [1,3]. The contributions are discussed here through three complementary perspectives: identifying and characterizing allergens, understanding factors that modify disease expression, and translating molecular information into clinical decisions. Together, they support an integrative model of precision allergology, in which molecular sensitization profiles are interpreted alongside exposure history, environmental context, cofactors, epithelial biology, and evidence of clinical reactivity.

## 2. Molecular Diagnostics in Allergen Identification and Characterization

Unexpected allergen sources can expose important diagnostic blind spots. Branicka et al. (Contribution 1) describe α-Gal sensitization in patients investigated for otherwise unexplained anaphylaxis, angioedema, or urticaria. The heterogeneous presentations, variable latency after meat consumption, and accompanying cofactors or comorbidities illustrate why a conventional search for an immediate food-related trigger may be insufficient. In this small series, clinical severity was not explained by the absolute α-Gal-specific IgE concentration, although a formal correlation could not be established. The editorial significance of these cases lies in the need to match molecular testing to a carefully reconstructed exposure history, rather than to interpret a positive result as a complete explanation of the reaction.

Cross-reactive allergen families present a different diagnostic challenge. In a prospective single-center study of Lithuanian adults with atopic disease, Sakalauskaite et al. (Contribution 2) identified sensitization to non-specific lipid transfer proteins (nsLTPs) in a selected cohort that included patients with systemic reactions of unknown origin. Discrepancies between extract-based and component-based results, particularly for Mal d 3 and Art v 3, highlight the limits of identifying an allergen source without resolving the proteins involved. Distinguishing sensitization to broadly cross-reactive nsLTPs from sensitization to other components within the same source may clarify complex clinical histories. However, these findings should be interpreted within the selected study population and do not establish the prevalence or clinical significance of nsLTP sensitization in the general population.

The increasing availability of multiplex assays also makes comparability between platforms a clinical concern. Majsiak et al. (Contribution 3) compared ALEX and ImmunoCAP ISAC in 40 Polish children hospitalized for food allergy and/or anaphylaxis. Across 3160 paired comparisons involving 79 allergen molecules shared by both platforms, qualitative results were concordant in 92.5% of comparisons. This overall agreement supports their use for sensitization profiling, while molecule-specific discrepancies caution against assuming equivalence for every allergen. Differences in assay design, including the use of a cross-reactive carbohydrate determinant (CCD) inhibitor in ALEX, also require consideration. Crucially, the absolute allergen-specific IgE values were not directly interchangeable: ALEX provides quantitative results in kUA/L, whereas ImmunoCAP ISAC reports semi-quantitative results in ISU. Thus, similar qualitative profiles do not imply equivalent numerical results or transferable clinical thresholds.

Together, these studies illustrate three complementary roles of molecular diagnostics: recognizing an overlooked allergen, resolving a cross-reactive sensitization pattern, and defining the limits of assay comparability (Contributions 1–3). Greater analytical resolution can improve the questions asked during clinical assessment, but it does not independently establish which sensitizations cause symptoms. Diagnostic thresholds developed for one platform therefore require validation before application to another, and every molecular profile remains anchored in the clinical history.

## 3. Towards Identifying Factors and Mechanisms Influencing Allergic Diseases

Environmental context provides an additional layer of interpretation. In their narrative review, Mazur and Czarnobilska (Contribution 4) discuss how air pollution and climate-related changes in pollen exposure may modify respiratory allergy, pollen allergenicity, and diagnostic interpretation. The evidence reviewed includes interactions between particulate matter and pollen allergens, enhanced basophil activation, and possible effects on AIT efficacy. These observations encourage assessment of the conditions under which allergen exposure occurs, while the clinical impact of specific pollutant–allergen interactions and their implications for treatment require further investigation.

Cofactors can similarly complicate the attribution of an allergic reaction to a single trigger. Rydzyńska et al. (Contribution 5) report anaphylaxis after grape consumption during ketoprofen use in a woman with broad nsLTP sensitization. The diagnostic work-up supported both nsLTP allergy and selective ketoprofen allergy, with tolerance to acetylsalicylic acid and ibuprofen. The case highlights the need to consider a non-steroidal anti-inflammatory drug both as a possible cofactor and as an independent culprit. Although one case cannot establish a general mechanism or estimate risk, it illustrates how molecular findings and a detailed drug history can guide a more discriminating diagnostic assessment.

At the tissue level, Yamamura et al. (Contribution 6) investigate extracellular ATP as a link between epithelial barrier dysfunction and inflammation in atopic dermatitis. In cellular experiments and an MC903-induced murine model, extracellular ATP reduced filaggrin expression and promoted thymus and activation-regulated chemokine (TARC) production. Inhibition of ATP release with clodronate reversed these changes and attenuated the disease-like phenotype. The study extends the Special Issue beyond sensitization profiling toward molecular mechanisms that may inform future treatment. Extracellular ATP is therefore a candidate therapeutic target, but the efficacy and safety of this approach in patients remain to be established.

These contributions place the allergen within a broader biological and exposure context: environmental conditions, concurrent drug use, and epithelial barrier function may all influence disease expression. Their evidence bases differ substantially, encompassing a narrative review, a single case report, and experimental models (Contributions 4–6). Their common contribution is to identify questions for targeted clinical investigation; they do not, by themselves, justify changes in routine treatment.

## 4. From Molecular Diagnosis to Clinical Decision-Making

The clinical usefulness of a molecular marker depends on whether it improves a decision that matters to the patient. In a narrative review of European pediatric studies, Balas et al. (Contribution 7) examine cow’s milk component-specific IgE as a predictor of oral food challenge (OFC) outcomes. Bos d 8 showed promising diagnostic performance, but its sensitivity and negative predictive value were insufficient to replace an OFC. Reported cut-offs varied across studies and age groups, and some cohorts found no advantage of individual components over whole cow’s milk-specific IgE. Bos d 8 is therefore best considered an adjunct to the assessment of challenge risk and changes in sensitization over time, rather than a universal threshold for confirming or excluding clinical allergy. The review reinforces the role of OFC, including the double-blind, placebo-controlled challenge as the diagnostic reference standard, when clinical reactivity remains uncertain.

Beyond its environmental perspective, the review by Mazur and Czarnobilska (Contribution 4) also illustrates how molecular diagnostics may inform therapeutic decision-making. CRD can help distinguish sensitization patterns and identify potentially relevant aeroallergens in polysensitized patients being considered for AIT. However, the selection of treatment still depends on a convincing relationship between allergen exposure and symptoms, and further trials are needed to establish the added clinical benefit of CRD-guided approaches. Functional assays such as the basophil activation test may provide additional information in selected settings, but limited availability and cost constrain their routine use.

The common principle is that molecular information should refine a clinical decision rather than substitute for evidence of clinical reactivity. In food allergy, component-specific IgE may inform whether and how to undertake a challenge, but it cannot replace an OFC in every patient whose diagnosis remains uncertain [1] (Contribution 7). In respiratory allergy, a molecular profile may guide allergen selection for AIT, while clinical relevance remains essential to treatment qualification [3] (Contribution 4). The next step is to demonstrate that this additional information improves diagnostic accuracy, treatment selection, or patient outcomes.

## 5. Conclusions

This Special Issue presents molecular allergology as an increasingly informative part of an integrated clinical approach. Its contributions show how component-level testing can reveal unexpected triggers, clarify cross-reactive sensitization, and support decisions about food challenges and immunotherapy. They also expose the limits of analytical detail: agreement between multiplex platforms does not make their numerical results interchangeable, and the detection of sensitization does not establish clinical allergy. Environmental exposures, cofactors, and epithelial biology provide essential context, while experimental work on extracellular ATP opens a possible avenue for therapeutic investigation. Future progress requires validation of diagnostic thresholds across populations and platforms, prospective evaluation of emerging mechanisms, and evidence that molecularly informed decisions improve outcomes. The central message is that precision allergology requires integration: molecular information gains clinical value when combined with phenotype, exposure history, cofactors, and validated assessment of clinical reactivity.
